# Supplementary material for: Hormone Receptor Expression in Meningiomas: A Systematic Review
Source: Cancers (Basel). 2023 Feb 3;15(3):980. doi: 10.3390/cancers15030980 (PMC9913299; doi:10.3390/cancers15030980)
Supplement: Supplementary file 1 [file cancers-15-00980-s001.zip › cancers-2132924-supplementary.pdf]

**Table S1.** All 61 included articles and their bias

* Intermediate

| Title and author | Year | Review | Bias Type |
|---|---|---|---|
| Li Q. et al. Emerging association between androgen deprivation therapy and male meningioma: significant expression of luteinizing hormone-releasing hormone receptor in male meningioma. | 2013 | Prostate Cancer Prostatic Disease | Strong |
| Carroll RS et al. Androgen receptor expression in meningiomas. | 1995 | Journal of Neurosurgery | Strong |
| Guevara P. et al. Angiogenesis and expression of estrogen and progesterone receptors as predictive factors for recurrence of meningioma. | 2010 | Journal of Neurooncology | Strong |
| Bozdağ M. et al. Association of apparent diffusion coefficient with Ki-67 proliferation index, progesterone-receptor status and various histopathological parameters, and its utility in predicting the high grade in meningiomas. | 2021 | Acta Radiologica | I* |
| Tao Y. et al. Clinical features and immunohistochemical expression levels of androgen, estrogen, progesterone and Ki-67 receptors in relationship with gross-total resected meningiomas relapse. | 2012 | British Journal of Neurosurgery | I* |
| Battu S. et al. Clinicopathological and molecular characteristics of pediatric meningiomas. | 2018 | Neuropathology | Strong |
| Barresi V. et al. Clinicopathological characteristics, hormone receptor status and matrix metallo-proteinase-9 (MMP-9) immunohistochemical expression in spinal meningiomas. Pathol Res Pract. | 2012 | Pathology research and Practice | Strong |
| Agaimy A. et al. Comparative study of soft tissue perineurioma and meningioma using a five-marker immunohistochemical panel. | 2014 | Histopathology | Strong |
| Telugu RB. et al. Estrogen and progesterone receptor in meningiomas: An immunohistochemical analysis. | 2020 | Journal of Cancer Research and therapy | I* |
| Bozzetti C. et al. Estrogen and progesterone receptors in human meningiomas: biochemical and immunocytochemical evaluation. | 1995 | Surgery Neurology | I* |
| Carroll RS. et al. Expression of a subset of steroid receptor cofactors is associated with progesterone receptor expression in meningiomas. | 2000 | Clinical Cancer Research | Strong |
| Carroll RS et al. Expression of estrogen receptors alpha and beta in human meningiomas. | 1999 | Journal of Neurooncology | Strong |
| Mnango L. et al. Expression of Progesterone Receptor and Its Association with Clinicopathological Characteristics in Meningiomas: A Cross-Sectional Study. | 2021 | World Neurosurgrery | I* |

| | | | |
|---|---|---|---|
| Schlegel J. et al. Expression of the c-erbB-2-encoded oncoprotein and progesterone receptor in human meningiomas. | 1993 | Acta Neuropathologica | Strong |
| Ihonen K. et al. Female predominance in meningiomas can not be explained by differences in progesterone, estrogen, or androgen receptor expression. | 2006 | Journal of Neurooncology | I* |
| Shahin MN. et al. Fertility treatment is associated with multiple meningiomas and younger age at diagnosis. | 2019 | Journal of Neurooncology | I* |
| Hirota Y. et al. Gonadotropin-releasing hormone (GnRH) and its receptor in human meningiomas. | 2009 | Clinical Neurology and Neurosurgery | Strong |
| Lusis EA. et al. High throughput screening of meningioma biomarkers using a tissue microarray. | 2005 | Journal of Neurooncology | Strong |
| Konstantinidou AE. et al. Hormone receptors in non-malignant meningiomas correlate with apoptosis, cell proliferation and recurrence-free survival. | 2003 | Histopathology | Strong |
| Perrot-Applanat M. et al. Immunocytochemical study of progesterone receptor in human meningioma | 1992 | Acta Neurochirurgica | Strong |
| Boulagnon-Rombi C. et al. Immunohistochemical Approach to the Differential Diagnosis of Meningiomas and Their Mimics. | 2017 | Journal of Neuropathology | I* |
| Brandis A. et al. Immunohistochemical detection of female sex hormone receptors in meningiomas: correlation with clinical and histological features. | 1993 | Neurosurgery | I* |
| Gursan N. et al. Immunohistochemical detection of progesterone receptors and the correlation with Ki-67 labeling indices in paraffin-embedded sections of meningiomas. | 2002 | International Journal of Neuroscience | I* |
| Takei H. et al. Immunohistochemical expression of apoptosis regulating proteins and sex hormone receptors in meningiomas.. | 2008 | Neuropathology | Strong |
| Leães CGS. et al. Immunohistochemical expression of aromatase and estrogen, androgen and progesterone receptors in normal and neoplastic human meningeal cells. | 2010 | Neuropathology | I* |
| Mezmezian MB. et al. Immunohistochemical Expression of Progesterone Receptors in Nonmeningothelial Central Nervous System Tumors. | 2017 | Applied Immunohistochemistry and Molecular Morphology | I* |
| Khalid H. et al. Immunohistochemical study of estrogen receptor-related antigen, progesterone and estrogen receptors in human intracranial meningiomas. | 1994 | Cancer | I* |
| Iplikcioglu. et al. Is progesteron receptor status really a prognostic factor for intracranial meningiomas? | 2014 | Clinical Neurological Neurosurgery | Strong |
| Heß K. et al. Brain Invasion in Meningiomas-Sex-Associated Differences are not Related to Estrogen- and Progesterone Receptor Expression. | 2017 | Neurosurgery | Strong |
| Ülgen E. et al. Meningiomas Display a Specific Immunoexpression Pattern in a Rostrocaudal Gradient: An Analysis of 366 Patients. | 2019 | World Neurosurgery | I* |

| | | | |
|---|---|---|---|
| Lusis EA. et al. Meningiomas in pregnancy: a clinicopathologic study of 17 cases. | 2012 | Neurosurgery | Strong |
| Maiuri F. et al. Meningiomas in Premenopausal Women: Role of the Hormone Related Conditions. | 2020 | Frontiers in Oncology | I* |
| Perry A. et al. Merlin, DAL-1, and progesterone receptor expression in clinicopathologic subsets of meningioma: a correlative immunohistochemical study of 175 cases. | 2000 | Journal of Neuropathology & Experimental Neurology | I* |
| Ichimura S. et al. Molecular investigation of brain tumors progressing during pregnancy or postpartum period: the association between tumor type, their receptors, and the timing of presentation. | 2021 | Clinical Neurological Neurosurgery | Strong |
| Portet S. et al. New Insights into Expression of Hormonal Receptors by Meningiomas. | 2020 | World Neurosurgery | Strong |
| Domínguez-Malagón HR. et al. Perineurioma versus meningioma. A multi-institutional immunohistochemical and ultrastructural studY. | 2021 | Ultrastructural Pathology | Strong |
| Hsu DW. et al. Progesterone and estrogen receptors in meningiomas: prognostic considerations. | 1997 | Journal of Neurosurgery | Strong |
| Pravdenkova. et al. Progesterone and estrogen receptors: opposing prognostic indicators in meningiomas. | 2006 | Journal of Neurosurgery | I* |
| Carroll RS. et al. Progesterone receptor expression in meningiomas. | 1993 | Cancer Research | Strong |
| Kuroi Y. et al. Progesterone Receptor Is Responsible for Benign Biology of Skull Base Meningioma. | 2018 | World Neurosurgery | I* |
| Wolfsberger S. et al. Progesterone-receptor index in meningiomas: correlation with clinico-pathological parameters and review of the literature. | 2004 | Neurosurgery Review | I* |
| Hua L. et al. Prognostic value of estrogen receptor in WHO Grade III meningioma: a long-term follow-up study from a single institution. | 2018 | Journal of Neurosurgery | I* |
| Bouillot P. et al. Quantitative imaging of estrogen and progesterone receptors, estrogen-regulated protein, and growth fraction: immunocytochemical assays in 52 meningiomas. Correlation with clinical and morphological data. | 1994 | Journal of Neurosurgery | Strong |
| Abdelzaher E. et al. Recurrence of benign meningiomas: predictive value of proliferative index, BCL2, p53, hormonal receptors and HER2 expression. | 2011 | British Journal of Neurosurgery | Strong |
| de Carvalho GTC. et al. Recurrence/Regrowth in Grade I Meningioma: How to Predict? | 2020 | Frontiers in Oncology | I* |
| Probst-Cousin S. et al. Secretory meningioma: clinical, histologic, and immunohistochemical findings in 31 cases. | 2003 | Cancer | Strong |
| Buhl R. et al. Secretory meningiomas: clinical and immunohistochemical observations. | 2001 | Neurosurgery | Strong |
| Baxter DS et al. An audit of immunohistochemical marker patterns in meningioma. | 2014 | Journal of Clinical Neuroscience | I* |

| | | | |
|---|---|---|---|
| Roser F. The prognostic value of progesterone receptor status in meningiomas. | 2004 | Journal of Clinical Pathology | I* |
| Claus EB, Park PJ. Specific genes expressed in association with progesterone receptors in meningioma. | 2008 | Cancer research | Strong |
| Smith JS. Sex steroid and growth factor profile of a meningioma associated with pregnancy. | 2005 | Canadian Journal of Neurological Sciences | Strong |
| Hatiboglu MA. Sex steroid and epidermal growth factor profile of giant meningiomas associated with pregnancy. Surg Neurol. | 2008 | Surgical Neurology | Strong |
| Nagashima, G et al. Immunohistochemical detection of progesterone receptors and the correlation with Ki-67 labeling indices in paraffin-embedded sections of meningiomas. | 1995 | Neurosurgery | Strong |
| Schulz, S et al. Immunohistochemical determination of five somatostatin receptors in meningioma reveals frequent overexpression of somatostatin receptor subtype sst2A. | 2000 | Clinical cancer research : an official journal of the American Association for Cancer Research | Strong |
| Menke, Joshua R et al. Somatostatin receptor 2a is a more sensitive diagnostic marker of meningioma than epithelial membrane antigen. | 2015 | Acta neuro pathologica | I* |
| Körner, Meike et al. Value of immunohistochemistry for somatostatin receptor subtype sst2A in cancer tissues: lessons from the comparison of anti-sst2A antibodies with somatostatin receptor autoradiography. | 2005 | The American journal of surgical pathology | I* |
| Lazow, Margot A et al. Immunohistochemical assessment and clinical, histopathologic, and molecular correlates of membranous somatostatin type-2A receptor expression in high-risk pediatric central nervous system tumors. | 2022 | Frontiers in oncology | I* |
| Behling, Felix et al. Differences in the expression of SSTR1-5 in meningiomas and its therapeutic potential | 2022 | Neurosurgical review | I* |
| Silva, Camila Batista de Oliveira et al. “Expression of somatostatin receptors (SSTR1-SSTR5) in meningiomas and its clinicopathological significance. | 2015 | International journal of clinical and experimental pathology | Strong |
| Barresi, V et al. Sstr2A immunohistochemical expression in human meningiomas: is there a correlation with the histological grade, proliferation or microvessel density? | 2008 | Oncology reports | Strong |
